# Supplementary figures and images for: Cancer and mortality risks among people with multiple sclerosis: A population-based study in Isfahan, Iran
Source: PLoS One. 2024 Oct 31;19(10):e0312707. doi: 10.1371/journal.pone.0312707 (PMC11527280; doi:10.1371/journal.pone.0312707)

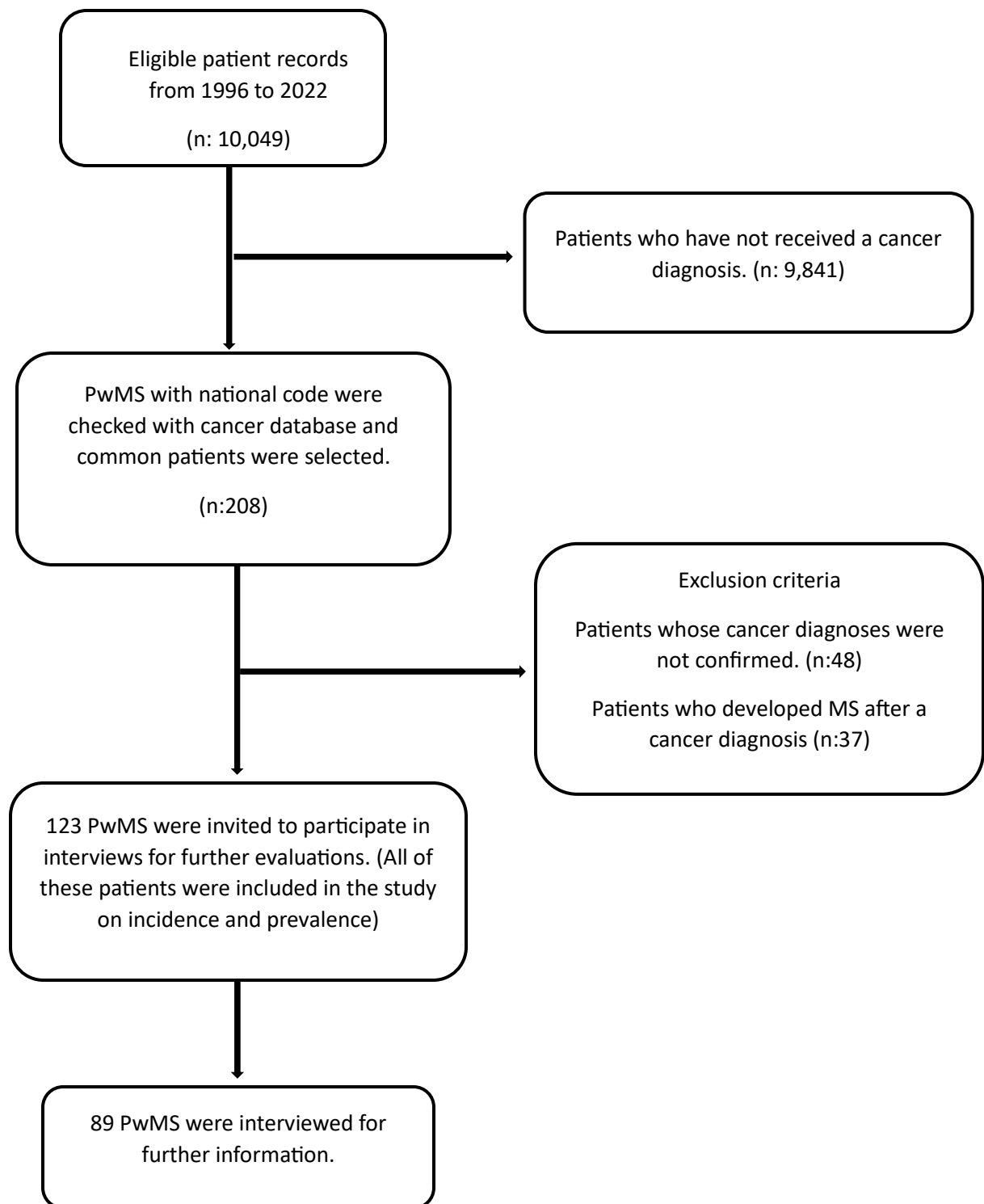

Figure S1: Flow diagram of the study process and participant selection

Supplement: S1 Fig — (PDF) [file pone.0312707.s001.pdf]
