# Supplementary material for: Cancer and mortality risks among people with multiple sclerosis: A population-based study in Isfahan, Iran
Source: PLoS One. 2024 Oct 31;19(10):e0312707. doi: 10.1371/journal.pone.0312707 (PMC11527280; doi:10.1371/journal.pone.0312707)
Supplement: S1 Table — (DOCX) [file pone.0312707.s002.docx]

**Supplementary Table 1**

| **Supplementary Table 1: Cancer incidence among People with Multiple Sclerosis** | | | |
| --- | --- | --- | --- |
| Incidence rate (95% CI) | Person-years at risk | Cancer (yes/no) |  |
| 125.6 (104.4-149.9) | 97925 | 123/9926 | All PwMS |
| Sex | | | |
| 123.1 (99.60-150.5) | 77168 | 95/7686 | Female |
| 134.9 (89.6-195.2) | 20757 | 28/2240 | Male |
|  |  |  | Age at MS |
| 94.49 (61.73-138.46) | 27514 | 26/2311 | <25 |
| 103.1 (64.6-156.1) | 21346 | 22/2078 | 25-29 |
| 140 (92.3-203.7) | 19282 | 27/2009 | 30-34 |
| 132.9 (77.5-212.9) | 12784 | 17/1414 | 35-39 |
| 115.3 (55.3-212.1) | 8674 | 10/971 | 40-44 |
| 170.3 (73.5-335.5) | 4698 | 8/577 | 45-49 |
| 353.2 (152.5-695.9) | 2265 | 8/304 | 50-54 |
| 303.3 (62.6-886.5) | 989 | 3/130 | 55-59 |
| 677.9 (82.1-2449.2) | 295 | 2/46 | 60-64 |
| - | 68 | 0/14 | 65-69 |
| - | 10 | 0/2 | 70-74 |
| - | - | 0/0 | 75-79 |
| - | - | 0/0 | +80 |

Abbreviation:MS ; Multiple sclerosis, PwMS ; People with MS
