# Supplementary material for: Cancer and mortality risks among people with multiple sclerosis: A population-based study in Isfahan, Iran
Source: PLoS One. 2024 Oct 31;19(10):e0312707. doi: 10.1371/journal.pone.0312707 (PMC11527280; doi:10.1371/journal.pone.0312707)
Supplement: S2 Table — (DOCX) [file pone.0312707.s003.docx]

**Supplementary Table 2**

| **Supplementary Table 2: Clinical characteristics of Multiple Sclerosis-Cancer patients by Survival Status** | | | | |
| --- | --- | --- | --- | --- |
|  |  | Overall | Survivors | Deceased |
| Age; mean (SD) | | 45.93 (11.58) | 46.13 (11.91) | 45.67 (11.23) |
| Sex; n (%) | Female | 95 (77.2) | 63 (88.7) | 32 (61.5) |
|  | Male | 28 (22.8) | 8 (11.3) | 20 (38.5) |
| Education years; mean (SD) | | 12.29 (3.89) | 12.41 (4.01) | 12.13 (3.75) |
| BMI; mean (SD) | | 24.70 (4.09) | 24.70 (4.09) | NR |
| Marital status; n (%) | Single | 5 (9.3) | 5 (9.3) | NR |
|  | Married | 47 (87) | 47 (87) | NR |
|  | Divorced | 2 (3.7) | 2 (3.7) | NR |
| Residence place; n (%) | Urban | 49 (90.7) | 49 (90.7) | NR |
|  | Rural | 5 (9.3) | 5 (9.3) | NR |
| Smoking; n (%) | No | 48 (88.9) | 48 (88.9) | NR |
|  | Yes | 6 (11.1) | 6 (11.1) | NR |
| Occupation; n (%) | Working | 15 (27.8) | 15 (27.8) | NR |
|  | Unemployed | 35 (64.7) | 35 (64.7) | NR |
|  | Retired | 3 (5.6) | 3 (5.6) | NR |
|  | Disabled | 1 (1.9) | 1 (1.9) | NR |
| Regularly exercise; n (%) | Yes | 10 (19.6) | 10 (19.6) | NR |
| Family income; n (%) | Lower class | 13 (25.5) | 13 (25.5) | NR |
|  | Middle class | 30 (58.8) | 30 (58.8) | NR |
|  | Upper middle class | 5 (9.8) | 5 (9.8) | NR |
|  | Upper class | 3 (5.9) | 3 (5.9) | NR |
| Diabetes; n (%) | Yes | 5 (9.3) | 5 (9.3) | NR |
| Hypertension; n (%) | Yes | 4 (7.4) | 4 (7.4) | NR |
| Hyperlipidemia; n (%) | Yes | 9 (16.7) | 9 (16.7) | NR |
| MS-associated parameters in MS-cancer patients | | | | |
| MS type; n (%) | RRMS | 99 (83.8) | 54 (81.7) | 45 (86.5) |
|  | SPMS | 18 (14.6) | 11 (15.5) | 7 (13.5) |
|  | PPMS | 2 (1.6) | 2 (2.8) | 0 (0) |
| MS Duration; mean (SD) | | 6.76 (3.91) | 5.62 (1.66) | 8.31 (5.33) |
| Age-onset-MS; mean (SD) | | 33.42 (10.83) | 33.61 (11.26) | 33.17 (10.31) |
| Baseline EDSS; median (Q_1_,Q_3_) | | 2 (1.5, 2.5) | 2.00 (1.5, 2.5) | NR |
| Current EDSS; median (Q_1_,Q_3_) | | 1.75 (0, 4) | 1.75 (0, 3.5) | NR |
| Family history of MS; n (%) | | 9 (16.7) | 9 (16.7) | NR |
| Family history of autoimmune; n (%) | | 6 (11.8) | 6 (11.8) | NR |
| First symptom; n (%) | Sensory | 13 (24.1) | 13 (24.1) | NR |
|  | Pyramidal | 14 (25.9) | 14 (25.9) |  |
|  | Optic | 19 (35.2) | 19 (35.2) |  |
|  | Cerebellar | 8 (14.8) | 8 (14.8) |  |
| DMT; n (%) | None | 5 (4.1) | 5 (7.0) | 0 (.0) |
|  | Interferon-β | 51 (41.5) | 25 (35.3) | 26 (50.0) |
|  | Monoclonal antibodies | 38 (30.8) | 24 (33.8) | 14 (26.9) |
|  | Teriflunomide | 10 (8.1) | 8 (11.3) | 2 (3.8) |
|  | Dimethyl fumarate | 8 (6.5) | 4 (5.6) | 4 (7.7) |
|  | Fingolimod | 5 (4.1) | 2 (2.8) | 3 (5.8) |
|  | Glatiramer acetate | 5 (4.1) | 2 (2.8) | 3 (5.8) |
|  | Azathioprine | 1 (0.8) | 1 (1.4) | 0 (0) |
| Cancer-associated parameters in MS-cancer patients | | | | |
| Age-onset-Cancer; mean (SD) | | 40.41 (11.64) | 41.14 (12.01) | 39.40 (11.15) |
| Survival time post-cancer diagnosis; mean (SD) | | 4.12 (2.45) | 5.62 (1.66) | 2.08 (1.80) |
| MS before cancer years; mean (SD) | | 7.00 (6.25) | 7.56 (6.94) | 6.25 (5.14) |
| Family history of cancer, n (%) | | 23 (45.1) | 23 (45.1) | NR |
| Cancer Type, n (%) | Breast (C50) | 31 (25.2) | 23 (32.5) | 8 (15.4) |
|  | Bone and articular cartilage (C40–C41) | 9 (7.3) | 2 (2.8) | 7 (13.5) |
|  | Digestive organs (C15–C24) | 17 (13.9) | 2 (2.8) | 15 (28.9) |
|  | Brain and other parts of central nervous system (C71–C72) | 12 (9.8) | 3 (4.2) | 9 (17.3) |
|  | Female genital organs (C51–C58) | 11 (8.9) | 10 (14.2) | 1 (1.9) |
|  | Secondary and unspecified sites (C77, C80) | 3 (2.4) | 1 (1.4) | 2 (3.8) |
|  | Prostate (C61) | 4 (3.3) | 3 (4.2) | 1 (1.9) |
|  | Connective and soft tissue (C49) | 2 (1.6) | 2 (2.8) | 0 (0) |
|  | Bronchus and lung (C34) | 3 (2.4) | 0 (0) | 3 (5.8) |
|  | Thyroid (C73) | 18 (14.6) | 17 (23.9) | 1 (1.9) |
|  | Urinary tract (C64–C67) | 6 (4.9) | 3 (4.2) | 3 (5.8) |
|  | Melanoma and other skin (C43–C44) | 7 (5.7) | 5 (7) | 2 (3.8) |
| Tumor grade, n (%) | 1 | 11 (8.9) | 9 (12.7) | 2 (3.8) |
|  | 2 | 11 (8.9) | 8 (11.3) | 3 (5.8) |
|  | 3 | 19 (15.5) | 14 (19.7) | 5 (9.6) |
|  | 6 | 1 (0.8) | 0 (0) | 1 (1.9) |
|  | 7 | 3 (2.4) | 0 (0) | 3 (5.8) |
|  | 9 | 78 (63.5) | 40 (56.3) | 38 (73.1) |
| Metastasis, n (%) | | 35 (28.5) | 21 (29.6) | 14 (26.9) |
| Tumor treatment, n (%) | Surgical | 51 (55.4) | 51 (55.4) | NR |
|  | Radiotherapy | 6 (6.5) | 6 (6.5) |  |
|  | Chemotherapy | 5 (5.4) | 5 (5.4) |  |
|  | Radiotherapy+ Chemotherapy | 18 (19.6) | 18 (19.6) |  |
|  | Radioactive iodine | 12 (13.1) | 12 (13.1) |  |
| Diagnostic Tests Tumor, n (%) | Biopsy | 71(47) | 71(47) | NR |
|  | PET scan | 15 (9.9) | 15 (9.9) |  |
|  | CT scan | 17 (11.3) | 17 (11.3) |  |
|  | Sonography | 26 (17.2) | 26 (17.2) |  |
|  | Mammography | 17 (11.3) | 17 (11.3) |  |
|  | Pap smear | 5 (3.3) | 5 (3.3) |  |
| Outcome | Living Recovered | 44 (35.7) | NR | |
|  | Living Under supervision | 7 (5.7) |  |  |
|  | Died | 52 (42.3) |  |  |
|  | Not responded | 20 (16.3) |  |  |

*Abbreviation: CT;* *Computed Tomography, PET; Positron Emission Tomography,BMI;Body mass index , DMT; Disease-Modifying Therapies , MS ; Multiple sclerosis , RRMS; Relapsing remitting MS, SPMS; Secondary Progressive MS, PPMS; Primary progressive MS*
