# Supplementary material for: Cancer and mortality risks among people with multiple sclerosis: A population-based study in Isfahan, Iran
Source: PLoS One. 2024 Oct 31;19(10):e0312707. doi: 10.1371/journal.pone.0312707 (PMC11527280; doi:10.1371/journal.pone.0312707)
Supplement: Attachment — Submitted filename: Comments .docx [file pone.0312707.s004.docx]

This manuscript titled; Cancer Risk and Mortality Among People with Multiple Sclerosis: A Population-Based Study in Isfahan, Iran.

Many patients with multiple sclerosis face numerous challenges associated with managing a chronic illness, which may lead them to overlook early warning signs that could indicate the presence of cancer. This oversight can result in delayed cancer diagnoses, potentially leading to a higher incidence of advanced-stage cancers and increased mortality rates.

This study aims to assist neurologists and specialists in MS by highlighting the potential link between cancer and MS, and by improving patient management strategies. While the discussion provided is thorough and relevant, it lacks specific guidelines for cancer screening in this patient population. Furthermore, given the higher prevalence of women with MS who are of childbearing age, the study could offer additional insights into the challenges faced by clinicians in treating MS and addressing reproductive health considerations, especially for breast cancer among women.

Considering the noted deficiencies, the article would benefit from minor revisions to address these challenges more effectively. Additionally, the language needs refinement.
