# Supplementary material for: Cancer and mortality risks among people with multiple sclerosis: A population-based study in Isfahan, Iran
Source: PLoS One. 2024 Oct 31;19(10):e0312707. doi: 10.1371/journal.pone.0312707 (PMC11527280; doi:10.1371/journal.pone.0312707)
Supplement: Attachment — Submitted filename: Comments.docx [file pone.0312707.s005.docx]

This article is well helped by the integration of MS and cancer registration systems to find the incidence rate, risk factors and mortality rate among MS patients in Isfahan.

But it has ambiguities that need to be resolved. Below are the comments:

**Title:** Mortality should be written as mortality rate (in title and whole body of manuscript).

**Abstract**

Abstract (Background): write the goal of study before method section.

Abstract (method): please specify deputy of MUIs and other registry systems such as NMSRI.

Abstract (method): data, collected following which international protocols?

Abstract (result): insert mean age instead of average age (Also in whole body of manuscript).

Abstract (result): put per 100,000 person-years at end of sentence.

Abstract (conclusion): write 2-3 sentences summarizing the supporting points / body paragraphs and delete Further research is needed …

**Introduction:**

Reference 1 in nor relevant for first paragraph.

Insert relevant reference for sentence “others indicate an increased or comparable risk”.

highest MS prevalence rates for Isfahan, needs relevant reference (no reference 11).

Describe the main goal of study in last sentence.

**Method:**

Please insert all data sources such as National MS Registry system, if you were used in this study.

Population of Iran is more than 85milion people, you should use last national census not 2016.

Specify data collection method from PwMS in manuscript with relevant references.

What is Vice-Chancellery of MUI,?

First, you must write the full word for the first time in the text and put its abbreviation in parentheses, like central nervous system (CNS). Follow this rule throughout the whole manuscript.

You should use latest McDonald criteria not 2001 version.

Insert exact number of excluded cases for each reason such as individuals who could not be identified through their national code, those who did not participate in follow-up interviews, those with insufficient data, or those whose cancer was diagnosed prior to the onset of MS.

How many subjects were collected for interview? While you mentioned about retrospective method and collecting data from registry system, why your team had interview with subjects?

How did you calculate EDSS by interview? It is not possible. Even types of MS.

This data is not exit in MUI registry system. It is available in National MS Registry of Iran.

Reference 18 is not suitable for this method.

**Result:**

Average ages should be change to mean age in whole manuscript.

Incidence ratio or ratio?

The overall incidence ratio of cancer among PwMS should be calculate correctly.

oldest age groups (65-79), should be write along with.

tumor grade 9? It should be corrected.

Last paragraph of “Clinical Features of Multiple Sclerosis-Cancer Patients” is confusing.

**Discussion**

Discussion is too long.

Insert relevant references for last paragraph of “Cancer Incidence Among People with Multiple Sclerosis: Higher or Lower?”

No need to discuses method of cancer diagnosis.

This study's strengths are not including analysis methods, detailed consideration of various influencing factors, and high ethical and methodological standards. It is necessary to do these things.

retrospective data from specific registries may

This study's limitation is not including introduce biases and inaccuracies, limiting the generalizability of findings beyond this population, absence of a control group from the general population and Retrospective data collection risks.

**Tables**

The total percentage of the columns in each category must be 100%. No less and no more.

What are the column titles in tables 2 and 3?

Write all Abbreviations bellow tables such as HR.

Why, apart from the references, the statistics of other variables have not been reported?

**Figure**

Specify cancer duration (Year).

**References**

References should be update and relevant to each sentence.

You should add NMSRIs references.

**The manuscript needs English editing.**
